# Metabolomic study combined with the low-level light therapy of Chinese acupuncture points and combined oral contraceptives in treatment of primary dysmenorrhea: A prospective, multicenter, randomized controlled study

**DOI:** 10.1016/j.heliyon.2023.e13821

**Published:** 2023-02-16

**Authors:** Hanbi Wang, Shiyang Zhu, Xuesong Ding, Yan Deng, Xiao Ma, Jingwen Gan, Yanfang Wang, Aijun Sun

**Affiliations:** Department of Obstetrics and Gynecology, National Clinical Research Center for Obstetric & Gynecologic Diseases, State Key Laboratory of Complex Severe and Rare Diseases, Peking Union Medical College Hospital, Chinese Academy of Medical Science and Peking Union Medical College, Beijing 100730, China

**Keywords:** Metabolomics, Primary dysmenorrhea, Low-level light therapy, Combined oral contraceptive, Biliverdin, Cortisol, Testosterone, Prostaglandin, Qihai (CV6), Guanyuan (CV4)

## Abstract

**Objective:**

To compare the changes of metabolites between Low-level light therapy (LLLT) and combined oral contraceptive (COC) after treatment of primary dysmenorrhea (PD), and to compare and analyze the biological and biochemical effects of the two treatments by analyzing the differences in metabolite profiles.

**Methods:**

A multicenter, double-blind, prospective, parallel, randomized controlled study was conducted on 69 women aged 16–35 years old with PD who were randomly divided into COC treatment group or LLLT treatment group. Low-level light therapy with light-emitting diodes (LED) was applied on two acupoints named “Guanyuan” (CV4) and “Qihai” (CV6). After 12 weeks of treatment intervention, blood samples were collected before and after treatment for metabolomic analysis. We used UPLC-MS/MS analysis to compare the differences in metabolite changes between LLLT and COC before and after treatment.

**Results:**

76 differential metabolites were detected in the LLLT group, and 92 differential metabolites were detected in the COC group, which were up-regulated or down-regulated (p < 0.001). Prostaglandin D2 (PG D2) was down-regulated and biliverdin was up-regulated after LLLT treatment, 4a-Hydroxytetrahydrobiopterin, Prostaglandin D2, 5-Hydroxy-l-tryptophan, Cholic acid were down-regulated and cortisol was up-regulated after COC treatment, and the differences were statistically significant. Cortisol and testosterone glucuronide in LLLT group were significantly lower than those in COC group. The metabolic pathways affected were glycerophospholipid metabolism, linoleic acid metabolism and arachidonic acid metabolism in the LLLT group, and glycerophospholipid metabolism, folate biosynthesis, arachidonic-acid-metabolism, and tryptophan metabolism in the COC group. The differential metabolic pathway were linoleic acid metabolism, steroid hormone biosynthesis, and alpha-Linolenic acid metabolism after the comparison of LLLT with COC.

**Conclusion:**

LLLT and COC might relieve dysmenorrhea by down-regulating PGD2, and LLLT might also relieve dysmenorrhea by up-regulating biliverdin. The level of cortisol and testosterone glucuronide after LLLT treatment was lower than that after COC treatment, which might lead to the difference in the clinical efficacy of the two treatments for dysmenorrhea.

## 1 Introduction

Dysmenorrhea is spasmodic pain originating from the uterus that may radiate to the thighs or lower spines, with lower abdominal pain, and may also be accompanied by discomfort such as nausea, headache, back pain, diarrhea, and fatigue during menstruation [1]. Dysmenorrhea is divided into primary dysmenorrhea (PD) and secondary dysmenorrhea. Primary dysmenorrhoea is pain in the absence of any organic cause and is characterised by cramping pain in the lower abdomen, starting within the first eight to 72 h of menstruation [2]. It is most common in adolescents and young adults [3]. According to data from the World Health Organization, its incidence varies from 45% to 97% in people of different ages and nationalities [4]. The pathogenesis of PD remains unknown, and excessive secretion of prostaglandins (PGs) is currently one of the widely accepted causes [1,5]. First-line drugs for the treatment of dysmenorrhea include nonsteroidal anti-inflammatory drugs (NSAIDs) and combined contraceptives (COC) [6]. However, long-term drug treatment may cause some adverse effects, such as the risk to the gastrointestinal tract, liver, kidney, and circulatory system by NSAIDs, and the risk of increasing thromboembolism by COC.

Low-level light therapy (LLLT) first came from Dr. Endre Mester, at the Semmelweis Medical University (Hungary) in 1967. This treatment method uses a low-power light source to promote tissue repair, decrease inflammation, and reproduce analgesia [7]. LLLT irradiation is used to stimulate uterine tissue cells, promote uterine cells to produce nitric oxide (NO), cyclic guanylate, and other substances [8], promote uterine smooth muscle soothing, restore the blood circulation of uterine tissue, enhance the metabolic effects of damaged uterine muscle cells. Through these biological activities, it finally achieves the purpose of relieving pain [9].

Metabolomics is a research method that shows the quantitative composition of low-molecular-weight compounds in a biological system. It represents the comprehensive metabolic status of samples at specific time points and is influenced by a variety of factors, including genetics, lifestyle, diet, drugs, exposure to toxins, and gut microbiota, in addition to the potential impact of diseases [10,11]. It has been increasingly used to identify metabolic biomarkers and improve the clinical diagnosis and treatment of diseases [12]. Metabolomics has many uses in the clinical field, including the study of disease mechanisms and biomarkers, disease diagnosis, inborn errors of metabolism testing, the impact of therapeutic interventions on patients and metabolism, efficacy, and safety of drugs [13]. The biological and biochemical effects of the treatment may be explained by analyzing differences in the metabolite profiles after therapeutic interventions (such as drug treatment).

In this study, LLLT combined with acupoint therapy of traditional Chinese medicine was used for the clinical treatment of PD. LLLT stimulated two major acupoints, “Qihai” (CV6) and “Guanyuan” (CV4), under the umbilicus, with COC treatment commonly used in clinical practice as the control group. Metabolomics research method was used to investigate the mechanism of action and efficacy material basis of LLLT in the treatment of primary dysmenorrhea, and compared with COC treatment.

## 2 Materials and methods

### 2.1 Study design

This study was a multicenter, prospective, parallel, randomized controlled study. Patients were recruited from gynecology clinics of 8 hospitals in 7 provinces of China from June 2019 to June 2020. This study was reviewed and approved by the Ethics Committee of Peking Union Medical College Hospital, Chinese Academy of Medical Sciences (Ethics Approval No. ZS-1913, Approval Date: 2019-03-26). It was also reviewed and approved by the ethics review boards of eight cooperating hospitals. The study protocol was registered and posted on ClinicalTrials.gov (NCT03953716, Registration Date: 2019-04-14, URL: https://register.clinicaltrials.gov/prs/app/action/SelectProtocol?sid=S0008TDC&selectaction=Edit&uid=U0003OAO&ts=5&cx=-dkue54). All study subjects signed the written informed consent form before starting the study. Because it was the first time to compare differences between COC and LLLT in PD treatment, we did not perform sample size calculation. To obtain reliable differences and standard deviations between the two groups, we included a larger number of cases than reported in previous studies.

### 2.2 Study population

A total of 135 healthy women aged 16–35 years old diagnosed with primary dysmenorrhea were included in this study.

#### 2.2.1 Inclusion criteria

It includes: (i) primary dysmenorrhea is defined as pain during the menstrual cycle in the absence of an identifiable cause [14]. (ii) Ultrasound and physical examination confirmed normal pelvic environment; (iii) Regular menstrual cycle (21–35 days); and (iv) Patients who can cooperate to complete the full cycle of treatment and follow-up as required.

#### 2.2.2 Exclusion criteria

It includes: (i) Patients who have had or have pelvic organic lesions that may cause secondary dysmenorrhea, including endometriosis, adenomyosis, pelvic inflammatory disease, adnexal cysts, space-occupying lesions, leiomyomas or endometrial polyp; (ii) Intra uterine device; (iii) Patients who are allergic to experimental drug components or have contraindications; (iv) Patients who have photosensitivity or skin lesions at the irradiation site; (v) Patients who have used trial-related drugs in the past 3 months and may interfere with the trial results; and (vi) Patients who have a thromboembolic disease or thrombosis tendency.

### 2.3 Study protocol

Patients included in the study were assigned to 2 groups, COC or LLLT, according to a random number table generated by a computerized SAS software (Cary, NC) block randomization technique, and both groups received 12-week treatment intervention. COC group received Marvelon (N.V.Organon, NL) as monophasic oral contraceptive formulation containing 30 μg of ethinyl estradiol and 150 μg of desogestrel (DSG/EE) in each tablet, from the fifth day of menstruation, one tablet per day for 21 days, followed by 7 days off and the next cycle of drug treatment.

**Fig. 1:**
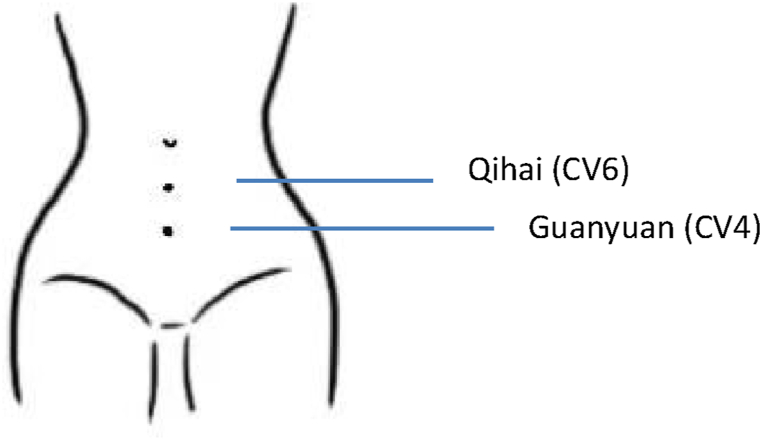
The location of the two acupoints where the pads should be positioned to CV6 and CV4. CV 6 is located on the medioventral line in the lower abdomen, 1.5 B-cun inferior to the centre of the umbilicus. CV4 is located 3 B-cun inferior to the centre of the umbilicus.

LLLT device, named Eospal (BEST Biotech Inc., CN), is specifically designed for self-management of dysmenorrhoea pain, approved by the China Food and Drug Administration (Registration No. 20182090168). It has been used clinically in recent years. The 630 nm wavelength (red light) and the power of 2.5 mW were applied to the two acupuncture points, named CV 6 and CV 4. CV 6 is the point on the Conception Vessel, located on the medioventral line in the lower abdomen, 1.5 B-cun inferior to the centre of the umbilicus. It can adjust the three yin meridians of the liver, spleen, and kidney, and is mainly used to treat urinary, reproductive, spleen and stomach diseases. CV4 is the intersection point of the Conception Vessel and three foot-yin meridians, which is located 3 B-cun inferior to the centre of the umbilicus and is used to treat abdominal distension, abdominal pain, etc. The LLLT group was treated with light irradiation for 20 min once every day for 5 days without interval for one week. During menstruation, the phototherapy was stopped. All the patients of the LLLT group received professional training to use the instrument correctly. Both therapies lasted for 12 weeks. To confirm the successful implementation of both treatments, the video examination in the LLLT group and pill count in the COC group were used, respectively. The detailed therapeutic method and illustrations about LLLT and the two acupoints had been presented in a published article [15], which was another part on the same subject. The clinical efficacy assessments for the COC group and LLLT group have been shown in the same article [15]. The locations of CV6 and CV4 were shown in Fig. 1.

Blood samples were collected at screening before treatment on day 2 of the menstrual cycle and at the visit of week 12 after treatment for metabolomics analysis.

A 10-cm visual analogue scale (VAS) ranging from 0 (no pain) to 10 (worst pain) was used for the pain grades at 10 a.m. on the first or second day of menstruation at each cycle (week 0, 4, 8, 12), in which mild pain was graded as 1–3, moderate pain 4–6 and severe pain 7–10.

### 2.4 Plasma samples preparation for metabolomics

Fasting blood samples were collected on the second day of the menstrual cycle at screening visit and week 12 after the treatment, and frozen immediately for analysis. Plasma samples were thawed at 4 °C on the ice. 100 μL plasma samples were accurately weighed, and the metabolites were extracted using a 400 μL volume of methanol: water (4:1, v/v) solution. After allowing to settle at −20 °C, the mixture was treated with high throughput tissue crusher Wonbio-96c (Shanghai wanbo biotechnology co., LTD) at 50 Hz for 6 min. The samples were then vortexed for 30s, sonicated with ultrasound at 40 kHz for 30 min at 5 °C, and placed at −20 °C for half hour to allow protein precipitation. Finally, the samples were centrifuged at 13000 g at 4 °C for 15 min and the supernatants were gently moved to sample vials for LC-MS/MS analysis.

### 2.5 Quality control sample

Initially, the performance of system conditioning and quality control processes required the preparation of a pooled quality control sample (QC) by mixing equal volumes of all samples. The testing of QC samples was conducted in a similar manner as the analytic samples. This practice facilitated signifying the whole sample set of samples (10 samples), which were injected at equal intervals with the aim to observe the analysis stability.

### 2.6 UPLC-MS/MS analysis

The metabolites were separated through chromatography using an ExionL CTMAD system (AB Sciex, USA) equipped with an ACQUITY UPLC BEH C18 column (100 mm × 2.1 mm i.d., 1.7 μm; Waters, Milford, USA). The volume and flow rate of sample injection were 20 μL and 0.4 mL/min, respectively. The temperature of the column was set at 40 °C. All the analyzed samples were stored at 4 °C.

The UPLC was attached with a quadrupole-time-of-flight mass spectrometer (Triple TOFTM 5600+, AB Sciex, USA) and an electrospray ionization (ESI) source. The ESI was operated in positive mode and negative mode. The optimal conditions followed in the process of analysis were as stated: source temperature, 500 °C; curtain gas (CUR), 30 psi; both Ion Source GS1 and GS2, 50 psi; ion-spray voltage floating (ISVF), −4000V in negative mode and 5000V in positive mode, respectively; declustering potential, 80V; a collision energy (CE), 20–60V rolling for MS/MS. Finally, data were acquired with Data Dependent Acquisition (DDA) mode. The sample detection was performed over a mass range of 50–1000 *m*/*z*.

### 2.7 Data preprocessing and annotation

After UPLC-TOF/MS analyses, the analysis peaks were detected from the raw data and aligned by using Progenesis QI 2.3 (Nonlinear Dynamics, Waters, USA). Metabolic characteristics assessed at least 75% in any set of samples were taken. The reproducibility was ensured by using the internal standard. The metabolic characteristics with the relative standard deviation (RSD) of QC>30% were abandoned. Mass spectra of these metabolic characteristics were recognized with the help of an accurate mass, MS/MS fragments spectra and isotope ratio difference with searching in reliable biochemical databases such as Human metabolome database (HMDB) (http://www.hmdb.ca/) and Metlin database (https://metlin.scripps.edu/). In a conclusive way, the mass tolerance between the measured *m*/*z* values and the exact mass of the ingredients of interest was ±10 ppm. For metabolites confirmed by using MS/MS, only components with MS/MS fragments score >30 were taken as definitely found values. If not, metabolites had only uncertain score.

### 2.8 Statistical and functional analysis

SPSS version 26.0 (IBM, Armonk, NY, USA) software was used for statistical analysis of the data. Clinical characteristics and treatment results of participants were compared using an independent sample *t*-test. Data are presented as means ± SD. Statistical significance was set at p < 0.05.

Umetrics SIMCA 14.1 software (Umeå, Sweden) was used to construct the partial least squares discriminant analysis (PLS-DA) and Orthogonal partial least squares discriminant analysis (OPLS-DA). PLS-DA is a useful approach to confirm the general separation of groups. OPLS-DA is an extension of PLS-DA and is helpful in distinguishing two groups and identifying different metabolites. Correlation analysis among QC samples was conducted by using ropls (Version1.6.2, http://bioconductor.org/packages/release/bioc/html/ropls.html) R package from Bioconductor on Majorbio Cloud Platform (https://cloud.majorbio.com). When analyzing differentially expressed metabolites, the criteria of both p-value <0.05 and fold change ≥1.5 (fold change is the ratio of the intensity of metabolite expression) were used. Pathway analyses were performed and visualized using the MetaboAnalyst 5.0 software package (https://www.metaboanalyst.ca/).

## 3 Results

We based our analysis on a random 69 sample of 135 participants. Clinical characteristics and therapeutic effect evaluation of dysmenorrhea: 36 patients in LLLT group and 33 patients in COC group were analyzed by metabolomics before and after treatment. The basic characteristics of patients of the two groups were balanced. VAS scores were significantly different between the two groups before and after treatment (p < 0.001), and the pain was significantly relieved after treatment, but there was no significant statistical difference between the groups, which were shown in Table 1.

**Table 1:** Clinical characteristics and treatment results of participants in this study (mean ± SD).

	LLLT（n = 36）	COC (n = 33)	*P* value
Age (years)	25.65 ± 4.60	25.72 ± 4.26	0.94
BMI	20.15 ± 2.94	19.88 ± 1.36	0.90
Age of menarche (years)	12.93 ± 1.36	12.96 ± 1.10	0.90
Menstrual cycle length (days)	29.58 ± 2.77	29.53 ± 2.52	0.93
Menstrual bleeding duration (days)	6.12 ± 1.25	6.12 ± 1.32	0.98
VAS before treatment	5.22 ± 2.59	5.39 ± 2.64	0.72
VAS after treatment	2.44 ± 1.79^a^	2.46 ± 1.05^a^	0.92

a Compared with VAS before and after treatment p < 0.001.

### 3.1 Overall results of metabolomics analysis

**Fig. 2:**
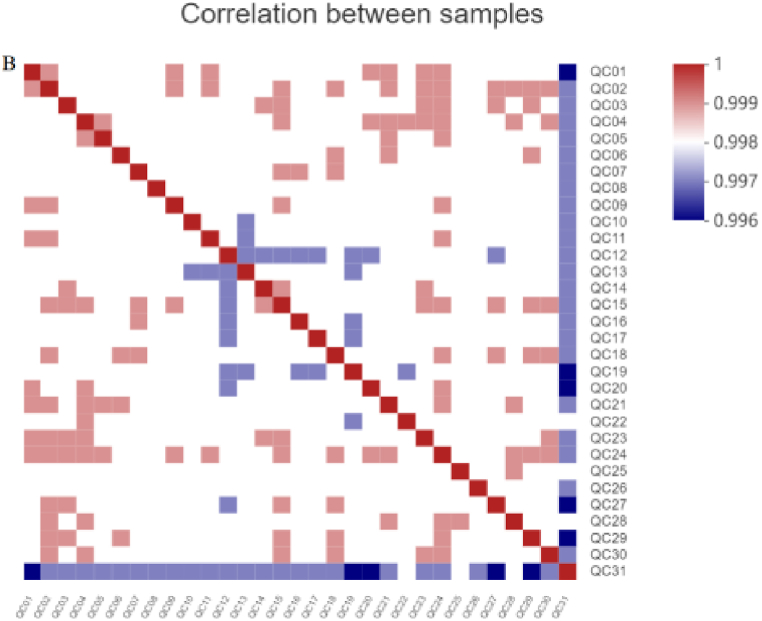
Correlation analysis among QC samples.

Firstly, 31 pooled samples were run as QC samples and the correlation analysis among QC samples was performed, which showed correlation coefficients ranging from 0.996 to 1 (Fig. 2), which indicated essential repeatability and stability throughout the analytical runs.

**Fig. 3:**
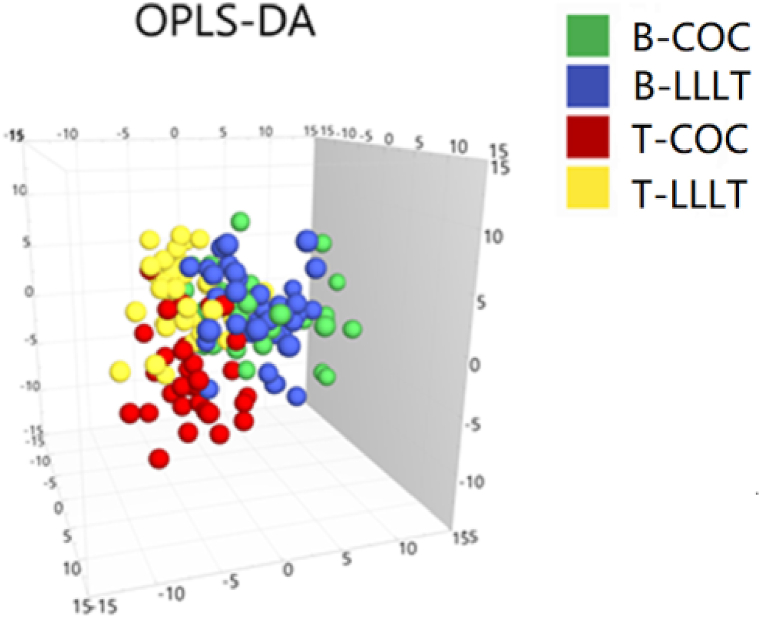
Metabolic plot of OPLS-DA in 4 groups, B: before treatment, T: treatment, COC: combined oral contraceptive, LLLT: low-level light therapy.

Then, a total of 861 metabolites were identified in four groups. 392 metabolites and 469 metabolites were identified in a positive mode in negative mode, respectively, which were shown in Table 2. To better show the difference between the four groups, a supervised OPLS-DA model was launched. The results showed a limited difference was found between the B-LLLT group and B–COC group, while the apparent separation between T-LLLT group and T-COC group, B-LLLT group and T-LLLT group, B–COC group and T-COC group were observed (Fig. 3).

**Table 2:** Metabolite identification results in four groups.

Ion mode	All peaks	Identified metabolites	Metabolites in library	metabolites in KEGG
Positive	5208	392	337	134
Negative	9013	469	433	107

### 3.2 Differential metabolites analysis

**Fig. 4:**
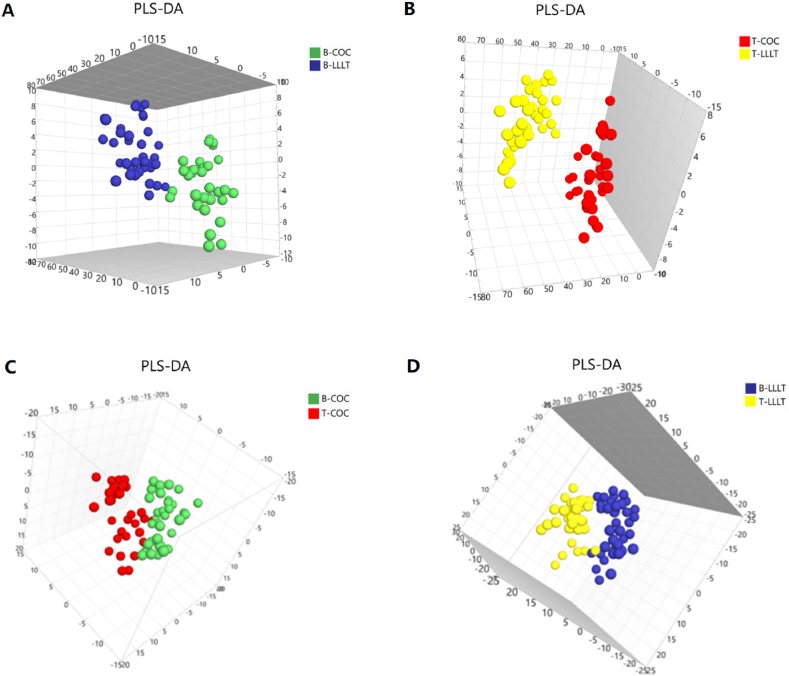
Metabolic plot of PLS-DA between B-LLLT and B–COC (A), T-LLLT and T-COC (B), B–COC and T-COC (C) and B-LLLT and T-LLLT (D). B: before treatment, T: treatment, COC: combined oral contraceptive, LLLT: low-level light therapy.

To identify the biomarkers between B–COC and B-LLLT, a supervised PLS-DA analysis was used to identify metabolic profiling differences. The results showed a significant difference between the two groups (Fig. 4A). Similarly, when comparing T-COC and T-LLLT, the PLS-DA analysis also showed an apparent distinction between the two groups (Fig. 4B). Then, the PLS-DA model was also used to analyze the treatment-associated metabolomic difference. In COC groups, PLS-DA analysis results showed a significant difference between the B–COC and T-COC (Fig. 4C). Then, PLS-DA analysis also showed a significant distinction between the B-LLLT group and T-LLLT group (Fig. 4D).

**Fig. 5:**
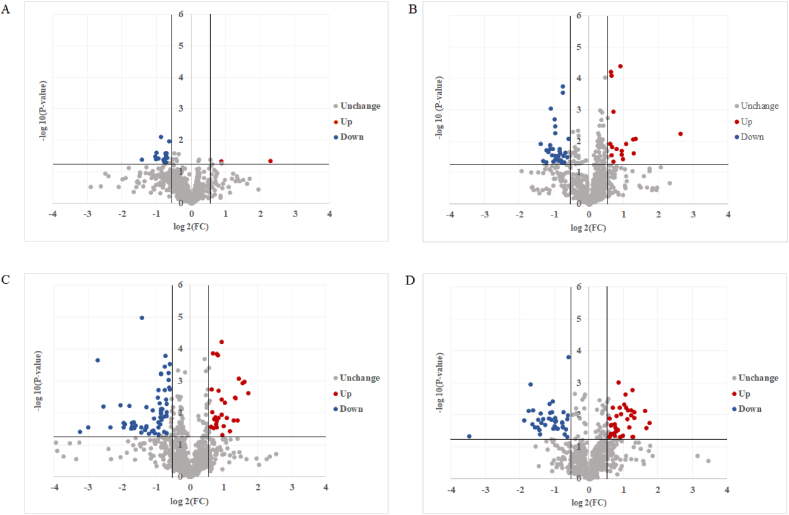
Volcano plots of differential metabolites between B-LLLT and B–COC (A), T-LLLT and T-COC (B), B–COC and T-COC (C), B-LLLT and T-LLLT (D).

Furthermore, based on the criteria of both p-value <0.05 and fold change ≥1.5, only 18 differential expressed metabolites were found between the B–COC group and the B-LLLT group (Fig. 5A). However, 48 significantly differential metabolites were identified between T-COC and T-LLLT, with 16 metabolites up-regulated in LLLT group and 32 metabolites down-regulated in (Fig. 5B). Then, significantly treatment-associated differences were found in both COC groups and LLLT groups. In COC group, a total of 92 significantly differential metabolites were found between B–COC and T-COC, with 30 metabolites up-regulated in T-COC and 62 down-regulated in T-COC (Fig. 5C). In LLLT group, 76 treatment-associated metabolites were obtained, compared with B-LLLT, 37 metabolites were found up-regulated and 39 were down-regulated in T-LLLT (Fig. 5D).

**Fig. 6:**
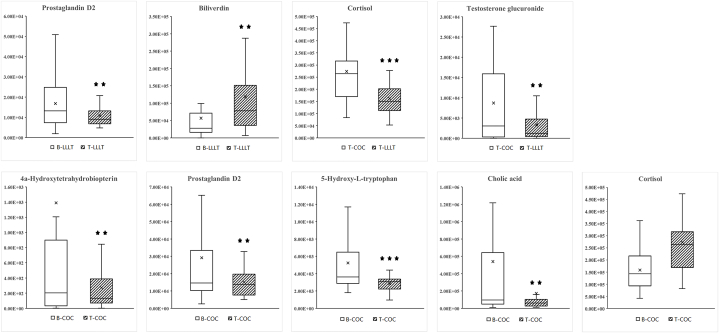
Relative abundance of differential metabolites (RA). **: p < 0.05; ***: p < 0.001; x: mean value.

Among the differential expressed metabolites in LLLT groups, prostaglandin D2 was found significantly down-regulated, while biliverdin was found significantly up-regulated after LLLT treatment. After COC treatment, 4a-hydroxytetrahydrobiopterin, prostaglandin D2, 5-hydroxy-l-tryptophan, and cholic acid were down-regulated and the difference was statistically significant. Cortisol was up-regulated after treatment, and the difference was statistically significant. The metabolites with statistically significant differences after COC and LLLT treatment were cortisol and testosterone glucuronide, which were significantly lower in the LLLT group than in the COC group, which were shown in Fig. 6.

### 3.3 KEGG analysis

**Fig. 7:**
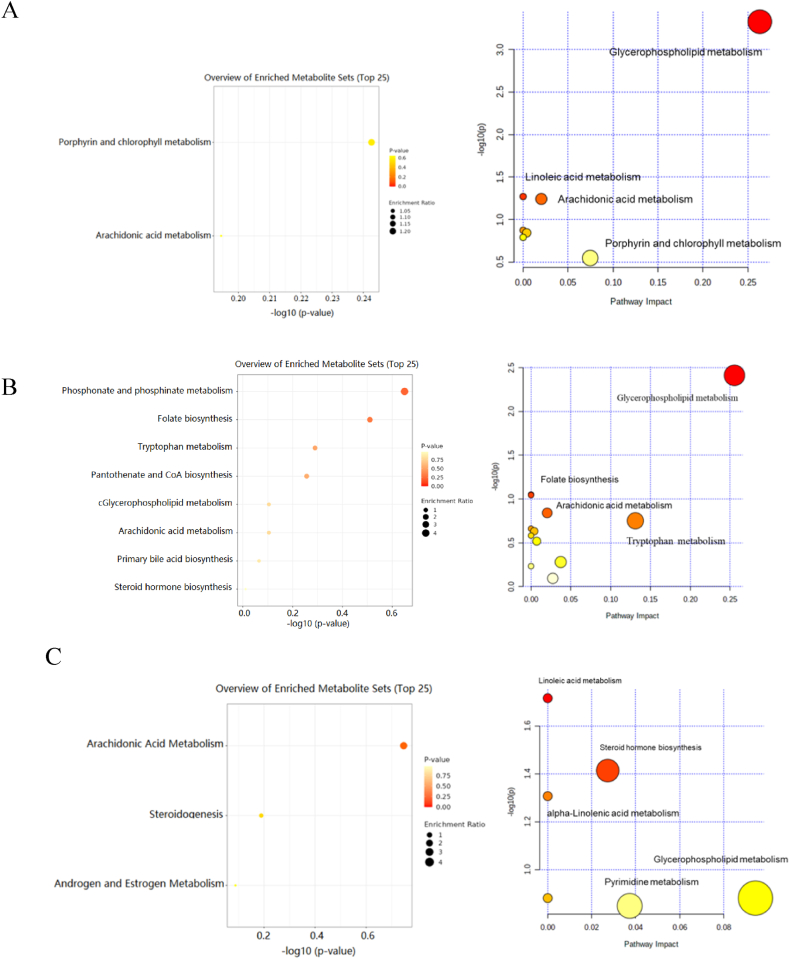
KEGG analysis. A. Pathway analysis of differential metabolites between B-LLLT group and T-LLLT group. B. Pathway analysis of differential metabolites between B–COC group and T-COC group. C. Pathway analysis of differential metabolites between T-LLLT group and T-COC group.

To further analyze the function of differential expressed metabolites, MetaboAnalyst (https://www.metaboanalyst.ca/) was performed. The function analysis results showed that LLLT treatment-associated metabolites were mainly associated the glycerophospholipid metabolism, Linoleic acid metabolism, and arachidonic acid metabolism (Fig. 7A), while the top four pathways of COC treatment-associated metabolites were glycerophospholipid metabolism, folate biosynthesis, linoleic acid metabolism, and arachidonic acid metabolism, which is shown in Fig. 7B. The differentially expressed metabolites between T-LLLT and T-COC were mainly related with linoleic acid metabolism, steroid hormone biosynthesis, alpha-Linolenic acid metabolism, which were shown in Fig. 7C.

## 4 Discussion

The results of this experimental clinical study have confirmed that both LLLT and COC treatment groups achieved the purpose of significantly relieving pain, and there was no significant difference in the effective rate between the two treatment methods. Both treatment methods reduced serum PGE2 levels to a similar extent [14]. The present study investigated the effect of LLLT treatment and COC treatment on the plasma metabolomics of patients with dysmenorrhea using LC-MS/MS. To our knowledge, this was the first study among plasma metabolomics studies to scan the influence of the two different treatments. According to the results, a significant metabolomics difference was shown in both LLLT group and COC group. Functional analysis revealed that most of the differential expressed metabolites were related to immunologic function and inflammatory response function, and so on [[16], [17], [18]].

Biliverdin levels were found to be significantly increased after LLLT treatment by metabolomics analysis. Previous studies have confirmed that biliverdin has anti-oxidative stress and free radical scavenging effects [19,20]. Ryan et al. reported that the initial inhibitory effect of biliverdin on the inflammatory cascade in the early phase of the inflammatory response may have reduced the degree of leukocyte infiltration, thereby promoting rapid resolution of inflammation. Its inhibitory effect on leukocyte infiltration is similar to the effect after ibuprofen treatment, confirming that biliverdin has an acute anti-inflammatory effect, which is similar to that of ibuprofen treatment [21]. biliverdin was found to protect rats from acetaminophen-induced toxicity [22], ameliorate cerebral ischemia-reperfusion injury [23], and inhibit the release of inflammatory cytokines in ischemia-reperfusion injury in rat studies, confirming that biliverdin can inhibit aseptic inflammatory response [24]. These studies all suggest the importance of biliverdin as an endogenous anti-inflammatory compound actively produced during aseptic inflammation and indicate that biliverdin may be an effective approach for the treatment of aseptic inflammation [21]. However, in the etiological study of dysmenorrhea, it was found that the concentrations of some inflammatory factors, such as prostaglandin F2α (PGF2α), tumor necrosis factorα (TNF), interleukin 6 (IL-6), C-reactive protein (CRP), vascular endothelial growth factor (VEGF) were increased in the menstrual cycle of dysmenorrhea patients [1,[25], [26], [27]]. Thus, we analyzed that LLLT may play an anti-inflammatory role by promoting the production of biliverdin, and then achieve the purpose of treating dysmenorrhea.

We also found that prostaglandin D2 levels were significantly lower in both COC and LLLT groups. Increased PG levels in dysmenorrhea patients are one of the widely accepted causes of PD, and PG triggers rhythmic uterine contractions, which reduce uterine blood perfusion and trigger pain [28]. Previous studies have shown that COC achieves the purpose of pain relief by inhibiting PG production and release [14], which is the same as our study results. After using LLLT to treat dysmenorrhea, we found that the efficacy of inhibiting PG could also be achieved by LLLT combined with acupoint therapy of traditional Chinese medicine, confirming the basis that LLLT could achieve the purpose of treating dysmenorrhea from the metabolite change level.

Arachidonic acid metabolism was found to be affected by both treatment methods after a comparative study between the COC group and LLLT group. Previous studies have shown that linoleic acid is catalyzed by specific dehydrogenases to produce Arachidonic acid, which is further catalyzed by cyclooxygenase (COS) to produce PG in cells [29,30]. COX is a key enzyme regulating prostaglandin release and includes two isoforms: structural cyclooxygenase-1 (COX-1) and inducible cyclooxygenase-2 (COX-2), of which COX-2 is an inducible form present only in inflammation and is considered a major source of inflammatory PG [31]. Both treatment methods may relieve dysmenorrhea by regulating Arachidonic acid metabolism to affect PG synthesis.

In comparison of COC with LLLT treatment, it was found that cortisol and Testosterone glucuronide (T-levels) were significantly higher in the COC group than those in the LLLT group, and the difference between the two groups was statistically significant. As early as 1975, studies have reported that plasma cortisol concentrations increased after COC used [32], and since then, COC has also been reported to increase cortisol levels [33,34]. Low cortisol levels or low response are associated with some chronic pain [35], including chronic pelvic pain (CPP) and irritable bowel syndrome (IBS) [36,37]. Animal studies have shown that the opioid response system for stress-induced analgesia is blocked by inhibition of the hypothalamic–pituitary–adrenal axis (HPA) [38], that is, repeated exposure to dysmenorrhea alters cortisol levels and cortisol stress response to the outside world [39]. Studies on cortisol and dysmenorrhea have confirmed that there is a significant negative correlation between the number of years of dysmenorrhea and cortisol levels, and the longer the number of years of dysmenorrhea is, the lower the cortisol levels are. Cortisol levels were not associated with the number of years of menstruation in women without dysmenorrhea [34].

For noxious stimuli, androgens act synergistically with estrogens to increase endogenous opioids release [40]. White and Robinson [41] found that low T-levels did not induce sufficient endogenous opioids to achieve pain suppression. Evans et al. investigated the relationship between T and dysmenorrhea and confirmed that there was a significant negative correlation between T-levels and dysmenorrhea [42]. Multiple mechanisms in the peripheral and central nervous systems have also demonstrated an inverse relationship between T-levels and pain [31]. Studies have shown decreased central brain activity associated with pain suppression in healthy women with low androgen levels [43]. A controlled study of COC in the treatment of dysmenorrhea found that there was a significant negative correlation between dysmenorrhea and androgen levels such as T, dihydrotestosterone (DHT), dihydroepiandrosterone (DHEA), and COC significantly increased sex hormone binding globulin (SHBG) levels and significantly decreased DHT, DHEA and free androgen index (FAI) levels, but had no significant effect on T, E2 or FEI [44,45]. Testosterone glucuronide is the major metabolite of testosterone and indirectly reflects T-levels. Our findings showed that COC had a stronger effect on cortisol and testosterone glucuronide compared with LLLT treatment, which may be related to pain scores after COC treatment [14]. The levels of cortisol and testosterone glucuronide after LLLT treatment were significantly lower than those after COC treatment, combined with previous studies, it could be seen that these two metabolites are related to relieve dysmenorrhea, and the different analysis from metabolomics might indicate that the effect of COC in relieving dysmenorrhea was better than that of LLLT. Although our clinical data do not show a statistically significant difference after treatment between the two approaches, expanding the sample size might obtain more accurate results.

Our study also found that LLLT and COC treatment had effects on different metabolic pathways. Glycerophospholipid metabolism and Arachidonic acid-metabolism were affected by both LLLT and COC treatment. LLLT affected Linoleic acid metabolism, and COC affected Folate biosynthesis and tryptophan metabolism, respectively. Further research is needed on the relationship between the changes in metabolic pathways and the therapeutic effect of dysmenorrhea, as well as the relationship between the difference in metabolic pathways and the difference in clinical efficacy after the two treatment methods.

## 5 Limitations of study

There were some limitations to this study: (i) Given the two treatment modalities were different, double blindness was impossible to carry out. Therefore, the patient's evaluation of the treatment effect might be affected. (ii) The combined treatment of acupoints and LLLT may play a role in promoting each other to improve the curative effect, and further study is needed on its mechanism. (iii) The functional explanation of differential metabolites is an inference based on the functional analysis of metaboanalyst and previous studies, which needs further research to confirm. (iv) At present, it is mainly focused to compare the metabonomic differences between the two treatment strategies, which cannot be used to assess the impact on the treatment effect. If there is a healthy control group, it might provide more information. (v) The sample size of the two groups was relatively small. More accurate conclusions could be obtained with a larger sample size.

## 6 Conclusion

In summary, LLLT and COC might relieve dysmenorrhea by down-regulating PG D2, and LLLT might also relieve dysmenorrhea by up-regulating biliverdin. The level of cortisol and testosterone glucuronide after LLLT treatment was lower than that after COC treatment, The relationship between the changes in metabolic pathways and clinical efficacy caused by LLLT and COC before and after treatment needs further study. The role of traditional Chinese medicine acupoints in the combined treatment with LLLT also deserves further study.

## Author contribution statement

Hanbi Wang: Conceived and designed the experiments; Performed the experiments; Analyzed and interpreted the data; Contributed reagents, materials, analysis tools or data; Wrote the paper.

Shiyang Zhu, Xuesong Ding, Yan Deng: Performed the experiments; Data collection.

Xiao Ma, Jingwen Gan, Yanfang Wang: Performed the experiments; Analyzed and interpreted the data; Wrote the paper.

Aijun Sun: Performed the experiments; Analyzed and interpreted the data ; Contributed reagents, materials, analysis tools or data.

## Funding statement

Dr Aijun Sun was supported by the National Natural Science Foundation (82074143) and the Capital’s Funds for Health Improvement and Research (CFH:2020-2-40113).

## Data availability statement

Data included in article/supp. material/referenced in article.

## Declaration of interest's statement

The authors declare no competing interests.

## Additional information

The clinical trial described in this paper was registered at ClinicalTrials.gov under the registration number NCT03953716.
